# Collective photonic response of high refractive index dielectric metasurfaces

**DOI:** 10.1038/s41598-020-72675-3

**Published:** 2020-09-24

**Authors:** Sushanth Reddy Amanaganti, Miha Ravnik, Jayasri Dontabhaktuni

**Affiliations:** 1 Mahindra Ecole Centrale, Hyderabad, India; 2 Faculty of Mathematics and Physics, University of Ljubljana, 1000 Ljubljana, Slovenia; 3 J. Stefan Institute, Jamova 39, 1000 Ljubljana, Slovenia

**Keywords:** Optics and photonics, Optical materials and structures, Metamaterials

## Abstract

Sub-wavelength periodic nanostructures give rise to interesting optical phenomena like effective refractive index, perfect absorption, cloaking, etc. However, such structures are usually metallic which results in high dissipative losses and limitations for use; therefore, dielectric nanostructures are increasingly considered as a strong alternative to plasmonic (metallic) materials. In this work, we show light-matter interaction in a high refractive index dielectric metasurface consisting of an array of cubic dielectric nano-structures made of very high refractive index material, Te in air, using computer modelling. We observe a distinct band-like structure in both transmission and reflection spectra resulting from the near-field coupling of the field modes from neighboring dielectric structures followed by a sharp peak in the transmission at higher frequencies. From the spatial distribution of the electric and magnetic fields and a detailed multipole analysis in both spherical harmonics and Cartesian components, the dominant resonant modes are identified to be electric and magnetic dipoles. Specifically at lower frequency (60 THz) a novel anapole-like state characterized by strong-suppression in reflection and absorption is observed, reported very recently as ‘lattice-invisibility’ state. Differently, at higher frequency (62 THz), strong absorption and near-zero far field scattering are observed, which combined with the field profiles and the multipole analysis of the near-fields indicate the excitation of an anapole. Notably the observed novel modes occur in the simple geometry of dielectric cubes and are a result of collective response of the metasurfaces. Periodicity of the cubic metasurface is shown as the significant material tuning parameter, allowing for the near-field and far-field coupling effects of anapole metasurface. More generally, our work is a contribution towards developing far-fetching applications based on metamaterials such as integrated devices and waveguides consisting of non-radiating modes.

## Introduction

Nanostructures with sub-wavelength periodicities and profiles are today explored as a major route for controlling the flow-of-light^1,2^. Metallic structures incident with plane electromagnetic waves in the sub-wavelength regime give rise to electromagnetic responses like effective negative refractive index^3^, perfect absorption^4^, perfect magnetic mirrors^5,6^, optical chirality^7^ and sub-diffraction limited imaging^8,9^. In such plasmonic materials, the dominant electro-magnetic modes giving rise to resonant behaviour are electric dipolar and quadrupolar modes whereas coupling to magnetic modes is weak, unless using specific material geometries to support the magnetic resonances like split-ring resonators (SRR’s). As an inherent challenge, these metallic-based materials suffer from losses due to field absorption resulting in heat dissipation. In contrast, dielectric and semi-conductor based photonic nano-structures unlike their metallic counterparts can overcome these losses and additionally also support magnetic resonance modes^10–12^. When electromagnetic wave is incident on the dielectric nano-structures, displacement currents are induced within the material giving rise to magnetic fields which resonate with the incident radiation to excite magnetic resonant modes naturally. Coupling of incident light with the oscillations of the phonon polaritons in the dielectric structures along with the near-field coupling between sub-wavelength structures hence give rise to pronounced magnetic-type resonances comparable to the electric resonances. Such magnetic dipolar resonances are observed recently in structures of various geometries like cylinders, disks, cubes and core-shell particles^13–20^ and further show novel applications such as perfect magnetic mirrors^5,6^, Huygens metasurfaces^21^and directional scattering^19,20,22^. Recently, a related work on optically resonant magneto-electric cubes shows that such structures can act as nanoantennas for ultra-directional light scattering^23^, showing impressive analysis of the scattering fields. In this context, our work focuses more on the detailed analysis of more complex resonant modes present in the system such as anapoles and the effect of collective response on such modes.

The resonance characteristics of the various electric and magnetic field modes present in a given system are generally determined by the geometry of the particles and the effective fill factor (i.e. the ratio between the lattice period and the size of the individual dielectric structures)^24,25^. A robust approach to solve such electromagnetic scattering problem of dielectric scatterers is to perform multipole response analysis, as was proposed in the Mie Scattering theory developed initially for spherical structures^10,18,26^. In Mie scattering approach, the field scattered by a single dielectric sphere in a homogeneous medium can be expanded into infinite series of vector spherical harmonics described by the electric and magnetic Mie coefficients, each coefficient representing the component of the respective multipolar modes. According to the Mie resonance theory^10^, the scattering properties of dielectric and non-magnetic materials depend on the dielectric permittivity $\epsilon$ of the scattering objects and the ratio of dimensions of the nano particle to the wavelength of the incident radiation known as the size parameter^11,12^. The dominant modes in the magnetic resonances are found to be of dipolar-type^13^ when the sizes of the meta-atoms are comparable to the incident wave length. It is also observed recently that as the refractive index of the material is increased, the field localization and the resonance characteristics are strongly pronounced within the dielectric structures^27^. Recently, anapoles and novel anapole-like states are observed in simpler dielectric geometries like cubes with very high refractive indices as a result of collective response of the metasurface^28–30^. The idea of this work is to investigate the field profiles inside the structures using the full wave electromagnetic simulations and multipole analysis, as well as to investigate the effect of periodicity of the metasurfaces on these novel modes.

In the present work, we study the electromagnetic response of a high-refractive index metasurface based on an array of sub-micron sized Te cubic structures to the incident frequency in 1–100 THz range. The collective response of the various field modes that are excited in the metasurface are investigated as a function of lattice constant of the dielectric metasurface. Specifically, we show a distinct band-like structure in the transmission and reflection spectra, which is caused by the near-field coupling between neighboring dielectric nanostructures. At higher frequencies, we also show the presence of novel toroidal modes with complete transmission and anapole states with very high absorption.

**Figure 1:**
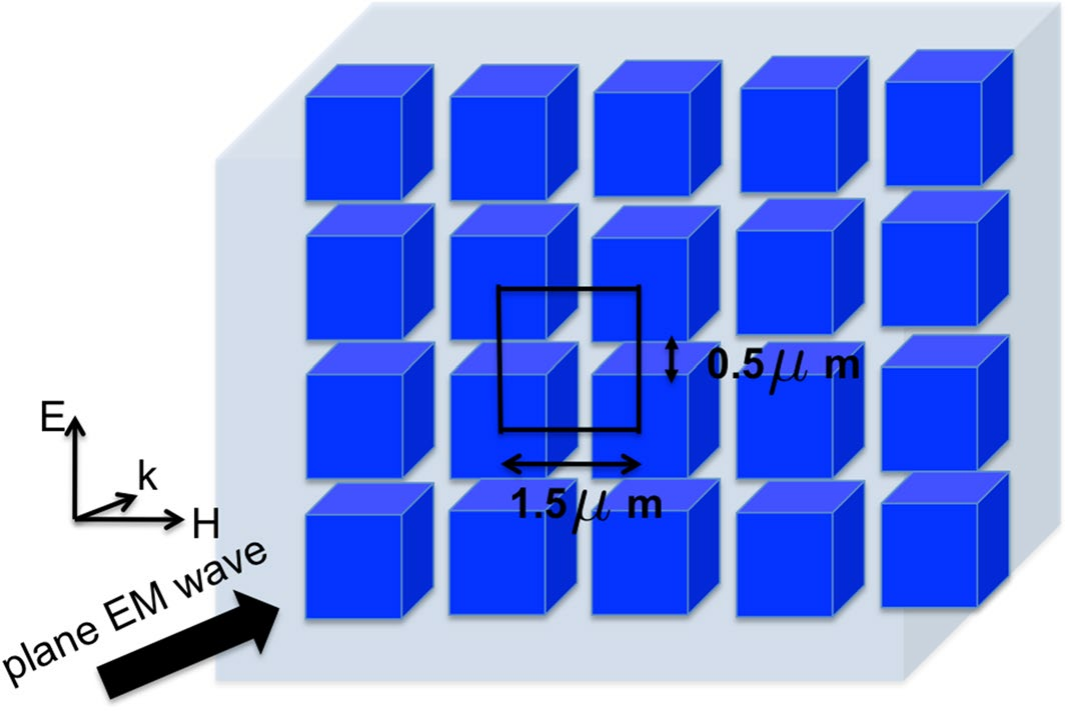
High refractive index dielectric metasurface consisting of Te $1\;\upmu {\text{ m }} \times 1\;\upmu {\text{ m }} \times 1\;\upmu {\text{ m }}$ cubic structures arranged in a periodic layer with in a cell of thickness $5\;\upmu$m. Square unit cell of the dielectric layer with lattice constant, $a=1.5\;\upmu$m is indicated in the figure.

**Figure 2:**
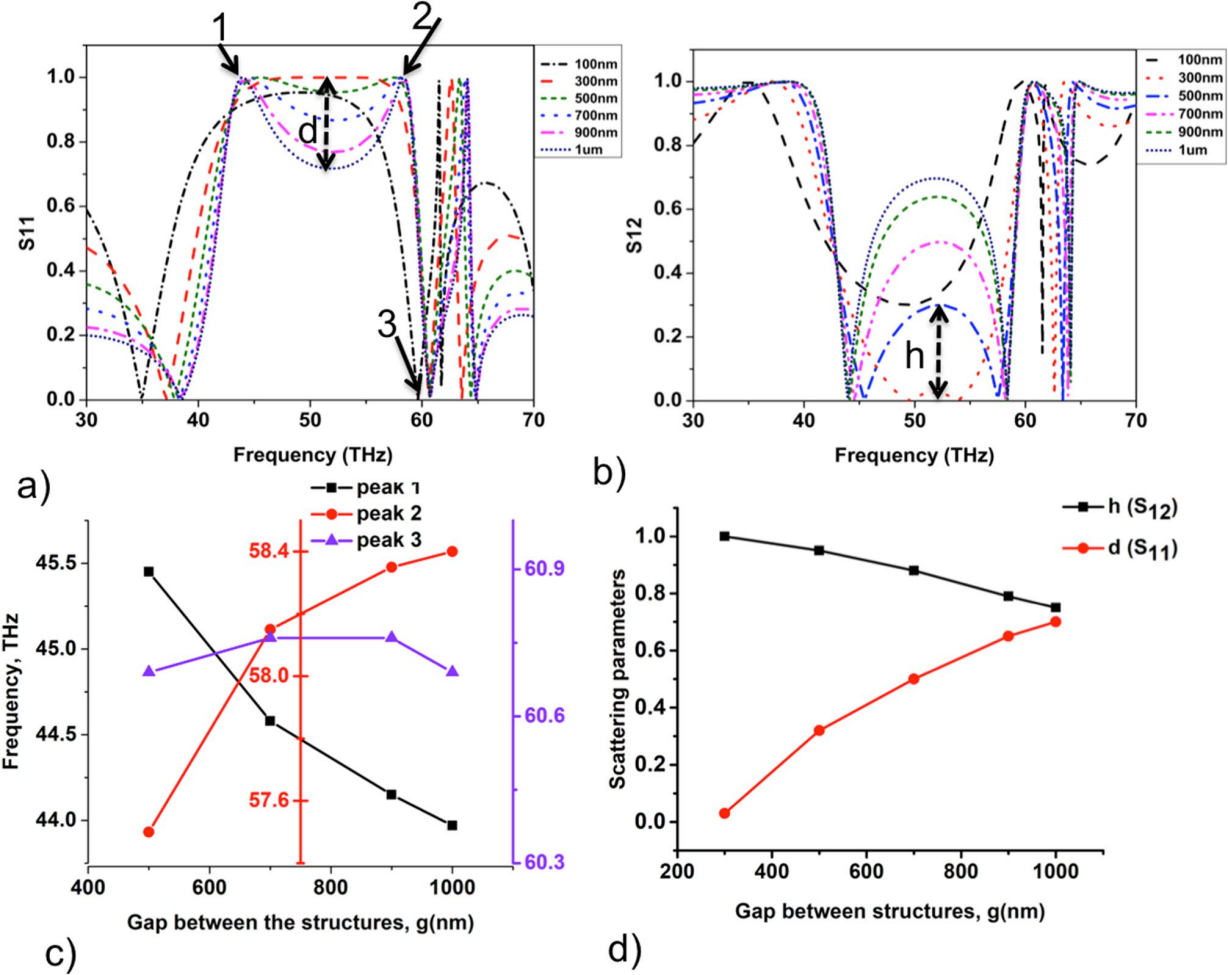
Transmission and reflection properties of the high refractive index Te metasurface. (**a**, **b**) Scattering parameters $S_{11}$ and (**b**) $S_{12}$ of the light scattered from the dielectric layer as a function of the frequency of the incoming light for different gap widths *g* between the structures respectively. (**c**) Resonance frequencies at 1,2 and 3, as indicated in (**a**), for $g=400$–1000 nm. (**d**) The height *h* and depth *d* of the separation in S parameters indicated in the (**a**) and (**b**) as a function of gap width *g* between the dielectric structures respectively.

## Design and simulation

Collective photonic response of the high refractive index nanostructures is determined by using numerical modelling, based on solving full Maxwell’s equations with finite element method. An array of cubic dielectric resonators (CDR) of dimensions $1\upmu {\text{ m }} \times 1\;\upmu {\text{ m }} \times 1\;\upmu {\text{ m }}$ is placed in XY plane, with the square unit cell of such resonator array shown in Fig. 1. Periodic boundary conditions are assumed within the XY plane directions, whereas in the *Z* direction the thickness of the simulation cell is set as $5\;\upmu {\text{ m }}$ (having embedded one layer of cubes in the middle). Electromagnetic plane waves in the frequency range 1–100 THz are incident normal to the layer of metasurface along the Z-direction with the incident electric field (polarisation) and magnetic fields along Y- and X-directions, respectively, as shown in the Fig. 1. Specifically the CST software is used to solve the electromagnetic equations in which the electric and magnetic fields are spatially discretized onto a lattice and Maxwell’s equations are solved using finite element method in frequency domain. This numerical method was used and well compared to experiments in multiple works^31–33^. Scattered waves from the dielectric structures are observed at the boundaries of the cell. The cubes of the metasurface are taken to be of high refractive index material Te with refractive index 5.7 (dielectric permittivity, $\epsilon =33.5$, permeability, $\mu =1$)^18^. We also carried out the simulations with materials Germanium, (n = 3.0) and Silicon (n = 2.0) to look at the effect of refractive index on the resonance characteristics. The response spectrum shifts to lower frequencies as the refractive index increases while qualitatively retaining the same spectral characteristics. In the present report, all the results presented are for Te cubic materials.

## Electromagnetic response and field profiles

### Scattering spectra and characteristic modes

We explore the electromagnetic response and field profiles of the high refractive index metasurfaces, by varying the incoming light frequencies and for different periodicities of the metasurface. Specifically, we perform simulations by varying the lattice constant, *a* of cubic Te dielectric structures in the range $a=1.1$–2 $\upmu$m keeping the dimensions of the particle to be same.

Complex scattering parameters $S_{11}$ and $S_{12}$ of the reflected and transmitted light from the dielectric metasurface are calculated in CST with results presented in Fig. 2. Scattering parameters are defined by $S_{11}=R$ and $S_{12}=Te^{(ik_0t)}$, where *R* and *T* are reflection and transmission coefficients of the material, $k_0$ is the wave number in free space and *t* is the thickness of the dielectric layer^34^. Figure 2a shows that for the frequencies of the incident light in the range $f=38$–60 THz (i.e., wavelength, $\lambda =3.5$–8 $\upmu m$), the S-parameters typically show a reflection band followed by a sharp peak in the transmission. For frequencies lesser than 38 THz ($\lambda > 8$ $\upmu$m) the incident electromagnetic waves undergo reflections at the boundaries of the cell giving rise to Fabry-Perot type oscillations. We study the effect of varying lattice constant on the response characteristics, scanning the ratios of $a=1.1$–$2\; \upmu$m. Figure 2a,b show the response spectra with the reflection band spanning the range 45–58 THz for $a=1.1$ $\upmu$m. As the lattice constant is increased the response band in the reflection and transmission spectra shifts towards higher frequencies and the band width increases, as seen in Fig. 2a,b. The response characteristics for the dielectric layer in the range of lattice constants $a=1.1$–$1.3\;\upmu$m corresponds to the regime in which the structures are close to each other such that the near-field coupling effects dominate the individual responses from the dielectric structures. This is evident from the characteristic flat bands in the S parameters, Fig. 2a,b. As the lattice constant is increased, the scattering coefficients show two distinct peaks emerging at the two ends of the band which separate into well-defined peaks with further increase in the lattice constant. The width of separation between the peaks increases with the increase in lattice constant as observed in Fig. 2c. The depth of separation in $S_{11}$ within the response band, *d* and the corresponding height of the separation, *h* in $S_{12}$ is observed to saturate for larger lattice constants as shown in Fig. 2c,d confirming the effect of near-field coupling for smaller lattice constants. Further, the resonance band is immediately accompanied by a sharp peak in transmission coefficient at 60.0 THz for all the lattice constants considered as shown in the Fig. 2a,b. The peak however widens slightly as the lattice constant is increased showing the signatures of the collective response for lesser lattice constants.

**Figure 3:**
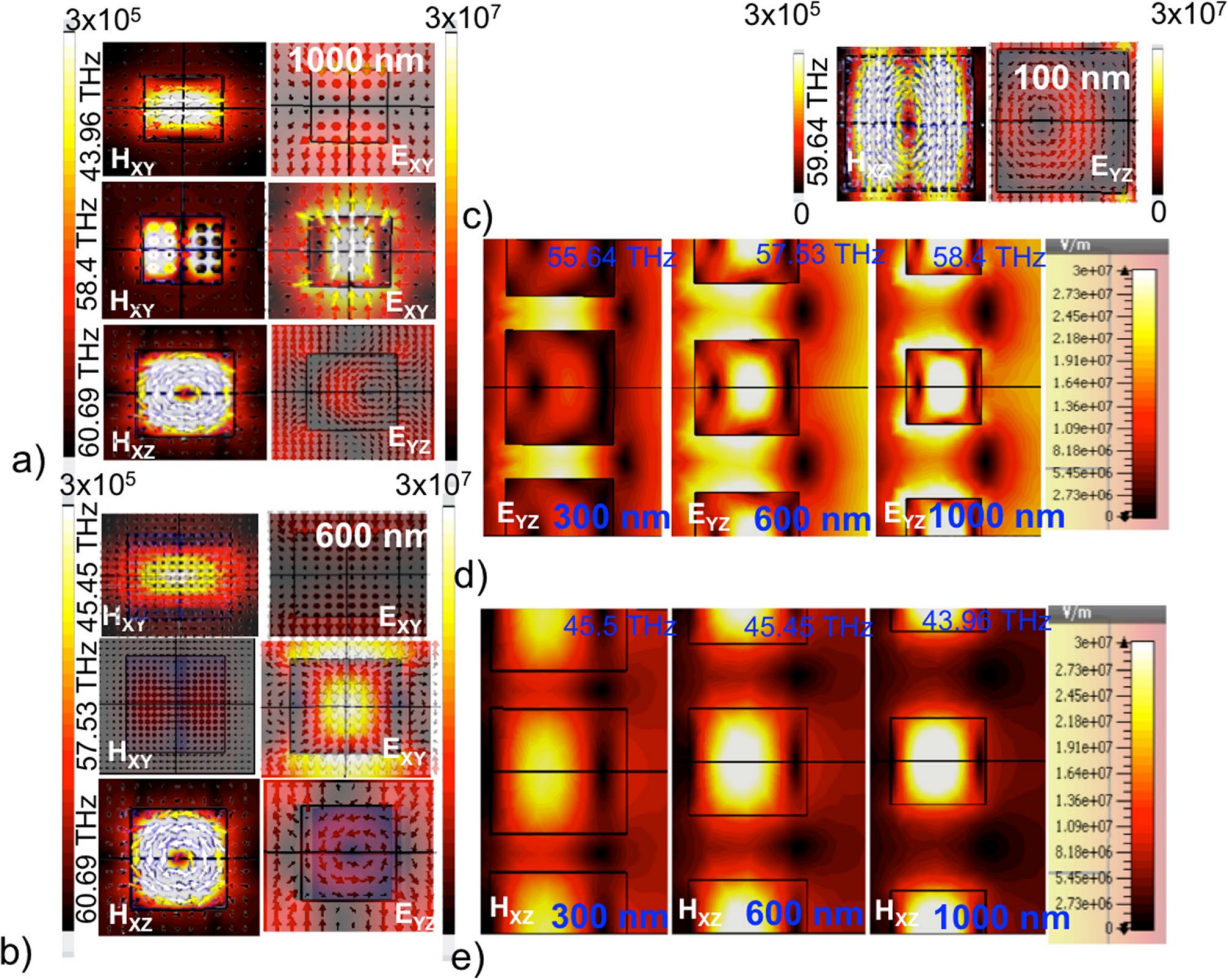
Resonant electromagnetic field modes. (**a**–**c**) The resonant field modes for lattice constants a = $2\;\upmu$m, $1.6\;\upmu$m and $1.1\;\upmu$m, respectively. The vectors indicate the direction of the corresponding fields in the planes indicated, whereas color indicates field magnitude. (**d**) Magnitude of the electric field in the YZ plane at the electric dipole resonance frequencies for lattice constants a = $1.3\;\upmu$m, $1.6\;\upmu$m and $2\;\upmu$m. (**e**) and the corresponding magnetic field in the XZ plane at magnetic dipole resonance frequencies respectively.

The collective response of the high refractive index dielectric structures to the incident light is clearly observed in the resonant field profiles (modes) within and surrounding the particles, as shown in Fig. 3a–c. In the case of smaller periodicities as seen for $a=1.1\;\upmu$m, the resonant mode occurs only at 60 THz, as a novel toroidal dipole mode. There are no resonant modes in the response band spanning the frequencies $f=35$–59 THz. In Fig. 3c, the toroidal mode is distorted in the case of $a=1.1\;\upmu$m due to neighboring mode-coupling. For periodicities above $a=1.3\;\upmu$m, the response spectrum shows resonances at the two ends of the Mie response band, corresponding to the excitation of the magnetic dipole at the lower frequencies and electric dipolar mode at the higher frequencies as shown in Fig. 3a,b. The fields are highly localized within the structure as seen from the figures. The toroidal dipole mode corresponding to the peak in the transmission exists for all the periodicities. As the lattice constant is increased to $a=1.5$ and higher, the distortion due to the interference effects from nearest neighbours reduces, giving rise to symmetric modes.

**Figure 4:**
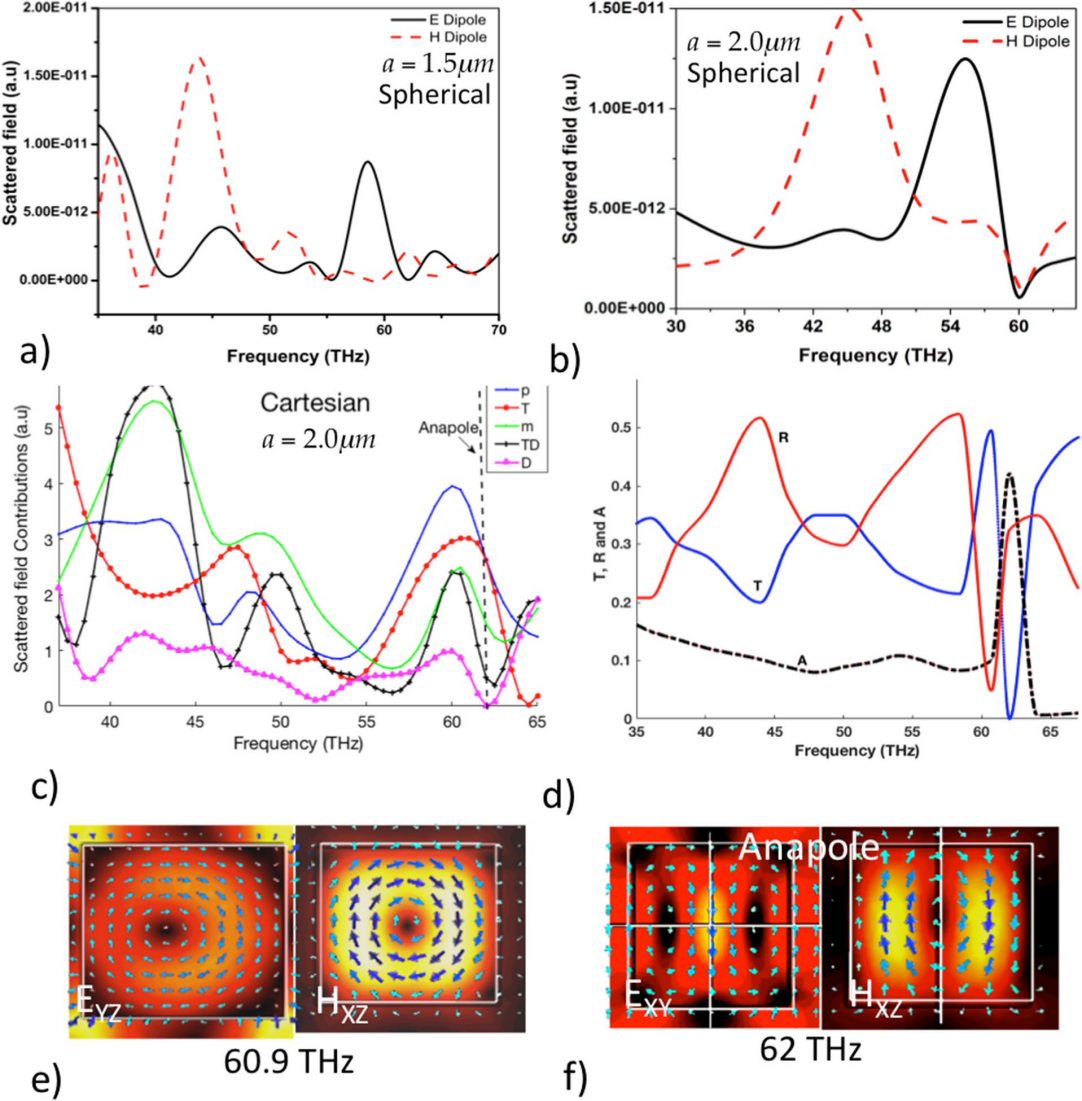
Analysis of the resonant modes. (**a**, **b**) Multipole coefficients of vector spherical harmonics for electric (black) and magnetic (red) dipoles scattered from the metasurface for lattice parameters $a=2.0$ and $1.5\;\upmu$m respectively, obtained at a distance of $r=1.2 r_0$, where $r_0$ is the radius of sphere circumscribing the cubes. (**c**) Normalized scattered field contributions of electric dipole (p), toroidal dipole (T), magnetic dipole (m), total electric dipole (D) and sum of all these contributions (TD) obtained from Cartesian decomposition for periodicity $a=2\;\upmu$m. (**d**) The intensity transmittance (T), reflectance (R) and absorbtance (A) obtained from electric and magnetic dipole contributions and (**e**, **f**) Magnitude and directions (represented as vectors) of field within the all-dielectric metasurface of the anapole-like state and anapole state, at 60 and 62 THz, respectively, for periodicity $a=2\;\upmu$m.

### Multipole analysis

Figures 3d,e show the effect of periodicity on magnitude of electric field and magnetic field distribution within the cubes at the left and right peaks of the response bands. At 300 nm, the coupling effects along the electric field are very high compared to the individual responses as seen from the high intensity fields between the structures. At 600 nm the field strength within and outside the structures are comparable and this corresponds to the cross-over from metamaterial regime to the diffraction regime. Above 600 nm, the fields are confined strongly within the structures and the coupling between neighboring structures is non-existent. Magnetic fields show a similar scenario at the resonance frequencies for magnetic dipolar modes but coupling between magnetic modes is less pronounced along X-direction.

Multipole analysis of the scattered fields from dielectric structures is a robust method to identify the resonant modes excited at various frequencies. Spherical multipole coefficients $a_E(l,m)$ and $a_M(l,m)$ are calculated by projecting the scattered fields $E_{sca}(r)$ obtained from the CST on a spherical surface (of radius $r_0$) enclosing the dielectric cube about a symmetric point (in this case centre of the cube) onto vector spherical harmonics using the relations below^35^:

1 $$\begin{aligned} a_E(l,m)= & {} \frac{(-i)^{l+1}kr}{h_l^{(1)} (kr)E_0\sqrt{\pi (2l+1)(l+1)l}}\int _0^{2\pi } \! \int _0^\pi \! Y_{lm}^*(\theta ,\phi ) E_{sca}(r)\sin \theta \,{\text {d}}\theta {\text {d}}\phi \end{aligned}$$

2 $$\begin{aligned} a_M(l,m)= & {} \frac{(-i)^{l}\eta kr}{h_l^{(1)}(kr)E_0\sqrt{\pi (2l+1)}} \int _0^{2\pi } \! \int _0^\pi \! Y_{lm}^*(\theta ,\phi ) H_{sca}(r)\sin \theta \,{\text {d}}\theta {\text {d}}\phi \end{aligned}$$

where $h_l$ are the spherical Hankel functions of first kind and $Y_{lm}(\theta ,\phi )$ are normalized spherical harmonics. *k* is the wave number of the incident wave in the surrounding medium whose impedance is given by $\eta$. Multipole decomposition in spherical harmonics given by the above equations allows for characterizing the optical properties in which multipoles of arbitrarily higher order are excited. Scattered fields can also be seen as originating from the displacement currents induced within the dielectric structures. The scattered field contributions from electric and magnetic dipoles for lattice constants $a=2.0$ and $1.5\;\upmu$m are plotted in the figures, Fig. 4a,b at a distance $1.2r_0$ where $r_0$ is the radius of the sphere circumscribing the cubic dielectric structure. The resonant peaks at left and right correspond to the H- and E-dipoles while the response band consists of non-resonant contributions from both the dipoles. For lower periodicities the coupling between electric and magnetic dipole modes is higher than for larger periodicities. In the case of $a=1.5\;\upmu$m as observed from Fig. 4b, there is strong suppression in both the electric and magnetic dipole contributions at 60 THz while for periodicity $a=2.0\;\upmu$m, there is a suppression in the electric dipole contribution at 62 THz. It is known that the scattered far-field from the metasurface can not distinguished in spherical harmonics whether originating from electric dipole or toroidal dipole. In order to investigate these individual contributions further we performed multipole decomposition of the scattered field in terms of displacement currents written in Cartesian coordinates^30,36^. Cartesian decomposition of the scattered field due to electric dipole, magnetic dipole and toroidal dipole contributions is given by:

3 $$\begin{aligned} \mathbf{E}_{sca}(r)=\frac{k_0^2}{4\pi \epsilon _0}\left\{ [\mathbf{n} \times (\mathbf{D} \times \mathbf{n})] + \frac{1}{v_d}[\mathbf{m}\times \mathbf{n}]\right\} \end{aligned}$$

where $\mathbf{D}=\mathbf{p}+i\frac{k_d}{v_d}{} \mathbf{T}$ is the total electric dipole moment where $\mathbf{p}$ and $\mathbf{T}$ are the electric dipole and toroidal dipole moment respectively. $\mathbf{m}$ is the magnetic dipole moment, $k_0$ is the wavenumber in vacuum, $k_d$ and $v_d$ are the wavenumber and the velocity of the light in the surrounding medium, respectively. $\mathbf{n}$ is the unit vector directed along $\mathbf{r}$. Hence the scattered electric field due to total electric dipole expanded in terms of electric dipole **p** and toroidal dipole **T** contributions is given by:

4 $$\begin{aligned} \mathbf{E}_{sca}(r)=\frac{k_0^2}{4\pi \epsilon _0} \left\{ [{\mathbf{n} \times (\mathbf{p} \times \mathbf{n})] + i\frac{k_d}{v_d} [\mathbf{n} \times \mathbf{T} \times \mathbf{n}}]\right\} \end{aligned}$$

where the cartesian component of the toroidal dipole contribution is given by $\mathbf{T}= \frac{1}{10c}\int {[(\mathbf{r}\cdot \mathbf{J})r-2r^2\mathbf{J}]{\text {d}}{} \mathbf{r}}$ and the electric dipole contribution $\mathbf{p}=\frac{1}{\omega }\int {\mathbf{J}{\text {d}}\mathbf{r}}$. The displacement current $\mathbf{J}$ is related to the electric field inside the dielectric structure as $\mathbf{J}= -i\omega \epsilon _0 (n^2-1) \mathbf{E}$^36^, *n* is the refractive index of the dielectric cube. Figure 4c shows the normalized scattered field contributions from E-dipole (**p**), H-dipole (**m**), toroidal dipole (**T**), total electric dipole (**D**) and sum of all the contributions (**TD**) calculated using cartesian decomposition for periodicity $a=2.0\;\upmu$m^30^. Toroidal contribution (**T**) is roughly comparable to the E-dipole (**p**) at the resonance frequency 60 THz where we observed near-zero reflection in the scattering parameters. Also, we observe a strong suppression in the TED contribution to the far-field at 62 THz with **T** and **p** being equal which is a strong indication of the anti-Kerker condition given by $\mathbf{p}=-ik\mathbf{T}$^37^. Anti-Kerker condition is satisfied when there is a destructive interference between the dipolar and toroidal modes giving rise to non-radiative states called anapoles^36,37^.

We further calculate the field transmission *t*, reflection *r* and absorption coefficients *a*^28^ from the total electric dipole, $\mathbf{D}$ and magnetic dipole components $\mathbf{m}$ for lattice constant $a=2\;\upmu$m. In our case, since the incident light is y-polarized, we assume $\mathbf{D}=(0,D_y,0)$, $\mathbf{m}=(m_x,0,0)$ and $\mathbf{n}=(0,0,n_z)$. Neglecting the higher order terms, the reflection and transmission coefficients are written as :

5 $$\begin{aligned} r= & {} \frac{ik_d}{2E_0L^2\epsilon _0\epsilon _d}\left[ p_{ty}+\frac{m_x}{v_d}\right] \end{aligned}$$

6 $$\begin{aligned} t= & {} 1+\frac{ik_d}{2E_0L^2\epsilon _0\epsilon _d}\left[ p_{ty}-\frac{m_x}{v_d}\right] \end{aligned}$$

The intensity transmittance, $T=|t|^2$, reflectance, $R=|r|^2$ and the absorbance $A=1-T-R$ are further used to analyse the field profiles. . At 60 THz a strong suppression in reflection, R is observed, see Fig. 4d, as also seen from the scattering parameters, Fig. 2a,b. This suppression in reflection accompanied by lower absorption and higher transmission indicates that the metasurface is highly transparent to the incident light, which is known as ‘lattice invisibility’^28^ due to generalized Kerker condition being satisfied. Figures 3 and 4e at 60 THz represent the fields in this novel anapole-like state which are asymmetric for smaller lattice constants. It is observed that the electric and magnetic fields circulate in toroidal fashion inside the structures as seen in Fig. 4e. However, it is interesting to note that resonance frequency of this state is independent of the lattice constant as seen from Figs. 2c and 3. At 62 THz there is a strong absorption and near-zero transmission, further confirming the enhanced field-localization within the dielectric particle, an indication for anapole, as also observed from the field profiles inside the dielectric structures, Fig. 4f. However, such states are not observed for periodicities $a=1.1$, 1.3 and $1.5\;\upmu$m considered in this work and the suppression of the total scattered field coincides with the ‘lattice-invisibility’ state at 60 THz as observed from Fig. 4b. This is due to the collective response of the metasurface which is higher at lower lattice constants. We also note that there is a reflection from the metasurface and the total scattered far-field is not zero, as ‘real’ anapole states have non-zero electric or magnetic field radiative modes present making it difficult to achieve completely non-radiative states with plane wave illumination^36^, while there are multiple attempts to achieve anapole states with structured light illumination^38,39^.

More generally, in the present work we observe the effect of collective response on anapole-like states at different metasurface lattice constants which is to the best of our knowledge not reported earlier in the literature. The high field-localization observed in the anapole states at 62 THz indicates interesting means for applications in sensing^40^ and imaging^41^ where strong magneto-electric field separations are desirable^42^. The high transparency of the metasurface at 60 THz and its independence of lattice constant could be used for possible applications in efficient sensing.

Also, we should comment that effective parameters of the metasurface including permittivity, permeability and refractive index can be in-principle retrieved from scattering parameters ($S_{11}$ and $S_{12}$)^34^. This retrieval method is quite widely used to extract effective parameters even for higher refractive index materials as seen in literature^43–45^; however, it should be noted that different retrieval methods and their implementation can lead to inconsistent data which violates Krammers–Kronig relations^46^.

## Discussion

Resonance spectra of high refractive index dielectric cubes made of Te in air are studied using CST based numerical simulations. In the sub-wavelength regime a characteristic response band is observed between Fabry–Perot oscillations at lower frequencies and higher order modes at the higher frequencies. The interference of the magnetic dipolar modes and electric dipole modes with comparable intensities result in this response band which is characteristic of high refractive index dielectric metasurfaces. The response band is followed by a resonance peak at 60 THz accompanied by strong suppression in reflection, which interestingly is independent of the lattice constant. Field profiles at this state indicate an anapole-like state with both electric and magnetic fields circulating within the dielectric structure which can be related to a state referred to as ‘lattice-invisibility’ state^28^. At slightly higher frequency 62 THz for larger lattice constants, anapole states are observed as a result of near-field coupling between toroidal and electric dipole modes. This anapole state exhibits strong electric and magnetic field-localization accompanied by high absorption and is the result of destructive interference between electric dipole mode and toroidal mode. Periodicity plays a significant role in observation of these novel modes. It is observed that the novel toroid state and anapole coincide for smaller periodicities while they occur at different frequencies for larger periodicities, establishing the role of collective response on the excitation of these states.

This work has potential applications in designing tunable anapole metasurfaces for applications in sensing and non-linear active metasurfaces with non-radiative components. So far, most of the studies on anapoles are investigated with disc geometries to sustain toroidal modes. Very recently^47^ authors investigated cuboid anapole metasurfaces as a function of the geometry. Our work opens up possibilities to study these anapoles with even simpler geometries by appropriate tuning of periodicity and refractive index of the dielectric.
